# Evaluation of the Antioxidant Activity and Antiproliferative Effect of the Jaboticaba (*Myrciaria cauliflora*) Seed Extracts in Oral Carcinoma Cells

**DOI:** 10.1155/2014/185946

**Published:** 2014-08-18

**Authors:** Wen-Hung Wang, Yu-Chang Tyan, Zong-Shiow Chen, Ching-Gong Lin, Ming-Hui Yang, Shyng-Shiou Yuan, Wan-Chi Tsai

**Affiliations:** ^1^Department of Otolaryngology, Cathay General Hospital, Taipei City 106, Taiwan; ^2^Department of Otolaryngology, Sijhih Cathay General Hospital, New Taipei City 221, Taiwan; ^3^School of Medicine, Fu-Jen Catholic University, New Taipei City 242, Taiwan; ^4^Department of Medical Imaging and Radiological Sciences, Kaohsiung Medical University, Kaohsiung 807, Taiwan; ^5^National Sun Yat-Sen University-Kaohsiung Medical University Joint Research Center, Kaohsiung 804, Taiwan; ^6^Translational Research Center, Kaohsiung Medical University Hospital, Kaohsiung 807, Taiwan; ^7^Institute of Medical Science and Technology, National Sun Yat-Sen University, Kaohsiung 804, Taiwan; ^8^Institute of Cosmetic Science, Chia-Nan University of Pharmacy and Science, Tainan 717, Taiwan; ^9^Instrument Technology Research Center, National Applied Research Laboratories, Hsinchu 300, Taiwan; ^10^Department of Medical Research, Kaohsiung Medical University Hospital, Kaohsiung 807, Taiwan; ^11^Department of Obstetrics and Gynecology, Kaohsiung Medical University Hospital, Kaohsiung 807, Taiwan; ^12^School of Medicine, College of Medicine, Kaohsiung Medical University, Kaohsiung 807, Taiwan; ^13^Department of Medical Laboratory Science and Biotechnology, Kaohsiung Medical University, Kaohsiung 807, Taiwan; ^14^Department of Laboratory Medicine, Kaohsiung Medical University Hospital, Kaohsiung 807, Taiwan

## Abstract

It is becoming increasingly evident that certain phytochemicals possess cancer chemopreventive properties. In this study, the antiproliferative activity of extracts from different parts of the jaboticaba (*Myrciaria cauliflora*) plant was evaluated for its effect on human oral carcinoma cell lines. The cytotoxicities of various plant extract concentrations were examined and the 50% maximal inhibitory concentration (IC_50_) was determined. Water extracts of jaboticaba seeds showed concentration-dependent antiproliferative effects. Annexin V/propidium iodide positivity with active caspase-3 induction indicated that the treated cells underwent apoptosis. Several important regulatory proteins (Bcl-2, Bcl-xL, Bid, and survivin) involved in apoptosis were also evaluated. The antioxidant activity of jaboticaba was investigated using 2,2-diphenyl-1-picrylhydrazyl (DPPH) and 2,2′-azinobis(3-ethylbenzothiazoline-6-sulphonic acid) (ABTS) assays, and the drug concentration eliciting 50% maximum stimulation (SC_50_) was determined. The present findings suggest that water extracts of jaboticaba seeds exhibit an antiproliferative effect against oral cancer cells by inducing apoptosis through downregulating survivin expression and thereby activating caspase-mediated Bid cleavage.

## 1. Introduction

Oxidation is essential to many living organisms for the production of energy to fuel biological processes. Reactive oxygen species are produced naturally in mammalian systems as a result of oxidative metabolism. Free radicals can lead to a variety of physiological and biochemical lesions and induce degenerative illnesses such as coronary artery disease and cancer [1]. Oxidative damage is balanced by endogenous antioxidants; however, the additional protection provided by nutritive and nonnutritive elements in food is critical for chemoprevention of diseases [2]. There has been increasing interest in finding naturally occurring antioxidants in foods and medicines to replace synthetic antioxidants, as their use is being restricted due to undesirable side effects [3].

Jaboticaba (*Myrciaria cauliflora*), belonging to the family Myrtaceae, is a purple-black plant with plum-sized fruit clusters that grow directly around the stem and main branches. It is known colloquially as the “Brazil grape tree” [4], is native to Brazil, and is distributed throughout the Atlantic Forest biome. The fruit has an appearance and texture similar to that of grapes, but with thicker, tougher, purple-colored skin. Each jaboticaba fruit contains one to four seeds in the white jelly-like flesh. Common in Brazilian markets, jaboticaba fruits are consumed fresh as well as in processed forms in jams, juices, and liqueurs. Their popularity has been likened to that of grapes in other countries [5].

Jaboticaba has been reported to be rich in phenolic constituents, including resorcinol, p-hydroxybenzoic acid, anthocyanins, hydroxycinnamic acids, flavonoids, coumarins, and ellagitannins. Phenolic compounds are well-known to be potent deactivators of reactive species, strong antioxidant and anti-inflammatory biological properties [5, 6], and anticancer potency [7, 8]. Gallic acid, one constituent of jaboticaba, a naturally occurring plant phenol, which can be abundantly found in natural plants, tea, and red wines [6, 9], has been demonstrated to have various biological properties, including antioxidant, anti-inflammatory, and anticancer activities [10]. Jaboticabin, a depside which is isolated from the fruit of jaboticaba, exhibits antiradical and anti-inflammatory activity and showed potential benefits in chronic obstructive pulmonary disease [5, 11]. A newly identified component of jaboticaba is syringin and its glucoside [6]. Syringin, the active principle of* Eleutherococcus senticosus*, can lower plasma glucose by increasing the release of acetylcholine from nerve terminals [12]. However, the antioxidant and anticancer potential of syringin remains to be elucidated.

Survivin is a member of the family of inhibitors of apoptosis proteins (IAPs) [13–15]. The integral role of survivin in cancer cell division and survival makes it an attractive therapeutic target to inhibit cancer cell growth [13–15]. It was originally suggested that survivin inhibits cell death induced through the extrinsic and intrinsic apoptotic pathways, conferring resistance to apoptosis by directly suppressing caspase activity [16]. Bid is a proapoptotic protein that upon activation by cleavage translocates to the mitochondria and binds there as a truncated Bid [17]. It can be cleaved by caspase-8 [14, 15], caspase-2 [18], and caspase-3 [19]. In the present study, we demonstrated that water extract of jaboticaba seeds exerts apoptotic activity on oral cancer cells through the suppression of survivin, thereby, activating caspase-mediated Bid cleavage.

## 2. Materials and Methods

### 2.1. Plant Extract Preparation

Jaboticaba fruits and stems were purchased from Tien-Yi Treegrape Farm in Chang-Hua County, Taiwan. The peels, stems, and seeds were isolated, washed, and air-dried thoroughly. For the ethanol-extraction part, peels, stems, and seeds were soaked in 95% ethanol with continuous shaking at room temperature for three days, respectively. The extracts were concentrated and strained through a 0.45 *μ*m filter, and the entire extraction process repeated twice. For the water-extraction part, the jaboticaba seeds were soaked in double-distilled water (ddH_2_O) at 50°C for three days and then concentrated and filtered. This water-extraction procedure was also performed twice.

### 2.2. 2,2-Diphenyl-1-picrylhydrazyl (DPPH) Assay

The antioxidant capacity was determined by the DPPH radical-scavenging method according to Brand-Williams et al. [20]. Various 100 *μ*L concentrations of the extracts in ethanol were added to 750 *μ*L 0.0025% ethanol DPPH solution. After a 30 min incubation period at room temperature, absorbance was read against a blank at 517 nm. DPPH is a purple-colored stable free radical which when reduced becomes yellow-colored diphenylpicrylhydrazine. The water-soluble vitamin E analogue 6-hydroxy-2,5,7,8-tetramethylchroman-2-carboxylic acid (Trolox) was used as a positive control. The inhibition ratio was calculated as % of inhibition using the following formula: [(absorbance of control − absorbance of test sample)/absorbance of control] × 100%. The extract concentration providing 50% inhibition (IC_50_) was calculated using a graph and plotting inhibition % against extract concentration [21].

### 2.3. 2,2′-Azinobis(3-ethylbenzothiazoline-6-sulphonic Acid) (ABTS) Assay

The free radical-scavenging activity of the examined essential oils was determined by the ABTS radical cation decolorization assay described by Pellegrini et al. [22]. ABTS radical cation was produced by reacting ABTS solution with 2.45 mM potassium persulfate (final concentration) and allowing the mixture to stand in the dark at room temperature for 12–16 h before use. The incubation mixture in a total volume of 5 mL contained 0.54 mL of ABTS radical cation, 0.5 mL of phosphate buffer, and varying concentrations of the extracts. Appropriate solvent blanks were run with each assay. The absorbance was read by spectrophotometer at 734 nm and compared with the Trolox control.

### 2.4. Cell Line Maintenance

The human oral squamous cell carcinoma cell line HSC-3 (Japan Health Science Research Resources Bank) was maintained in Dulbecco's modified Eagle's medium (DMEM) supplemented with 10% FBS. The cells were maintained in the appropriate growth medium at 37°C in a humidified atmosphere of 5% CO_2_ and 95% air and used over a restricted culture period of 10 passages.

### 2.5. Cell Viability Analysis

The effect of test extracts on cell viability was assessed using the 3-(4,5-dimethylthiazol-2-yl)-2,5-diphenyl-2H-tetrazolium bromide (MTT) assay in four to six replicates. HSC-3 cells were grown in 10% FBS-supplemented DMEM, in 96-well plates for 24 h, and then exposed to various concentrations of extracts in the same medium for predetermined time intervals. Controls received dimethyl sulfoxide (DMSO) and ddH_2_O vehicle at a concentration equal to that in drug-treated cells. At the end of the treatment, the medium was removed and replaced with 200 *μ*L of 0.5 mg/mL MTT in the same medium. Cells were then incubated in a CO_2_ incubator at 37°C for 2 h. Supernatants were removed from the wells and the reduced MTT dye was solubilized in DMSO 200 *μ*L/well. Absorbance was determined at 595 nm using a plate reader.

### 2.6. Annexin V/Propidium Iodide (PI) Staining and Flow Cytometry Analysis

After treating with extracts for 3 h, 5 × 10^5^ cells were collected and washed in cold phosphate-buffered saline (PBS). The supernatant was aspirated, leaving the pellet undisturbed. The procedure for staining with the ApoAlert Annexin V Kit (Clontech Laboratories Inc., Mountain View, CA, USA) was based on the manufacturer's protocol. In brief, the cells were resuspended in 100 *μ*L binding buffer. To each tube, 5 *μ*L Annexin V-fluorescein isothiocyanate (Annexin V-FITC, 20 *μ*g/mL) and 10 *μ*L PI (50 *μ*g/mL) were added. Each tube was then gently mixed and incubated at room temperature for 15 min in the dark. Cells were analyzed with a FACScan flow cytometer (Becton, Dickinson and Company, Palo Alto, CA, USA).

### 2.7. Caspase-3 Activity Assay

Caspase-3 activity was determined using the BD ApoAlert Caspase-3 assay according to the manufacturer's instructions (Becton, Dickinson and Company) as previously described. In brief, cell lysates were mixed with 10 mM dithiothreitol- (DTT-) rich reaction buffer containing 50 *μ*M DEVD-pNA, a caspase-3 substrate, and incubated for 1 h at 37°C. Enzyme-catalyzed release of pNA was monitored using a microplate reader at 405 nm. A total of 10,000 events were acquired for each sample and analyzed with a FACScan flow cytometer.

### 2.8. Protein Extraction and Western Blot Analysis

Biomarkers of apoptosis were assessed by Western blotting as follows. Treated cells were washed in PBS, resuspended in sodium dodecyl sulfate (SDS) sample buffer, sonicated for 5 s, and then boiled for 5 min. After brief centrifugation, equal amounts of total protein from each sample were fractionated by sodium dodecyl sulfate polyacrylamide gel electrophoresis (SDS-PAGE) and transferred to a polyvinylidene difluoride membrane. The transblotted membrane was washed thrice with Tris-buffered saline (TBS) containing 0.05% Tween 20 (TBST). After blocking with TBST containing 5% nonfat milk for 60 min, the membrane was incubated with an appropriate primary antibody at 1 : 1000 dilution in TBST-5% low-fat milk at 4°C overnight and then washed thrice with TBST. The membrane was probed with goat anti-rabbit or anti-mouse IgG-horseradish peroxidase conjugate (1 : 10000) for 1 h at room temperature and washed thrice with TBST. The hybridized immunocomplex was detected with Renaissance Chemiluminescence Reagent Plus (NEN Life Science Products, Boston, MA, USA). The quantitative analysis of Western blotting was carried out using the ImageQuant-TL-7.0 software, version 2010 (Amersham Biosciences).

### 2.9. Statistical Analyses

All analyses were run in triplicate and expressed as mean ± standard deviation (SD). Statistical significance was evaluated by Student's *t*-test and confidence limits were set at *P* < 0.05.

## 3. Results

### 3.1. Scavenging Activity of Jaboticaba

**Figure 1:**
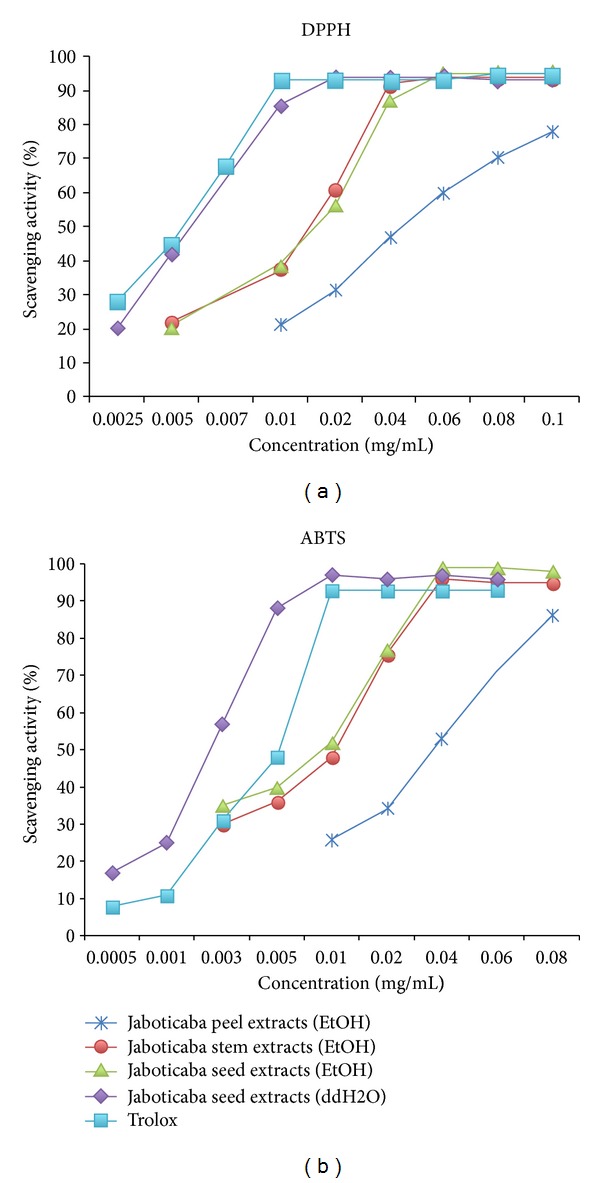
Antioxidative activity of different parts of jaboticaba.Using DPPH and ABTS assays, the water extract of jaboticaba seeds was found to have the best scavenging activity compared to other portions of the plant. Trolox served as a positive control.

The results of the determination of antioxidant activity of different extracts using two methods (DPPH and ABTS) are presented in Figure 1. For DPPH scavenging activity the water extract of jaboticaba seed was more active than the ethanol extract of seed and other parts of jaboticaba. The SC_50_ of ethanol extracts of peel, stem, seeds, and water extract of seeds were 0.049, 0.017, 0.027, and 0.0059, respectively. The SC_50_ of water extract of seed was close to the positive control, Trolox (0.0053).

In addition, water extract of the seed showed much more active antioxidant potential in ABTS scavenging. The SC_50_ of ethanol extracts of peel, stem, and seed and water extract of seed were 0.038, 0.01, 0.091, and 0.0027, respectively, whereas the SC_50_ of Trolox was only 0.0052. These data indicate that the water extract of seed possesses very potent antioxidant activity.

### 3.2. Antiproliferative Activity of Plant Extracts from Different Parts of Jaboticaba

**Figure 2:**
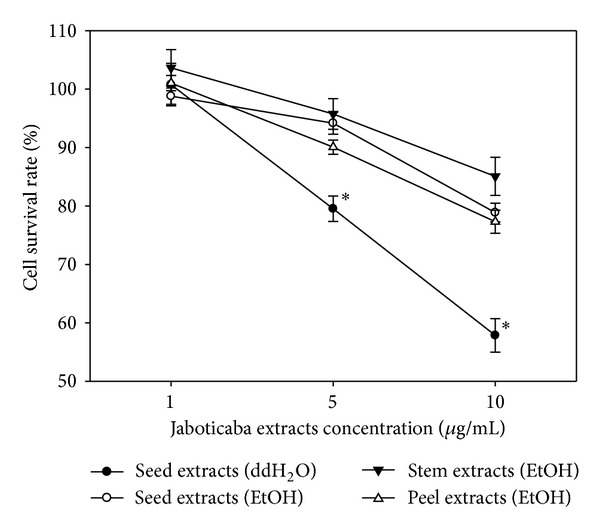
Water extract of jaboticaba seeds possessed the most potent cytotoxic activity. Cell survival rate of HSC-3 cells after 24 h treatment with various extracts showed that the water extract of jaboticaba seeds had the most significant cytotoxic activity compared to extracts from other portions of the plant. The IC_50_ of water extract of jaboticaba seeds in HSC-3 cells was approximately 15 *μ*g/mL. Each point represents the mean and SD of six determinations.

To evaluate which part of jaboticaba possesses the most potent antiproliferating effect on oral cancer cells, we treated HSC-3 cells with jaboticaba water or ethanol extracts from different parts of the plant. Cytotoxicity on HSC-3 cells determined using the MTT assay is shown in Figure 2. Of the four types of extracts (jaboticaba seed/water, seed/ethanol, stem/ethanol, and peel/ethanol), only seed/water showed a marked inhibition effect on HSC-3 cell viability and it was in a dose-dependent manner. After a 24 hr treatment, the IC_50_ of jaboticaba seed/water extract was approximately 15 *μ*g/mL. These data suggest that the seed/water extract of jaboticaba is the most potent of all of the extracts we examined.

### 3.3. Induction of Apoptosis in Jaboticaba Seed Extract-Treated HSC-3 Cells

**Figure 3:**
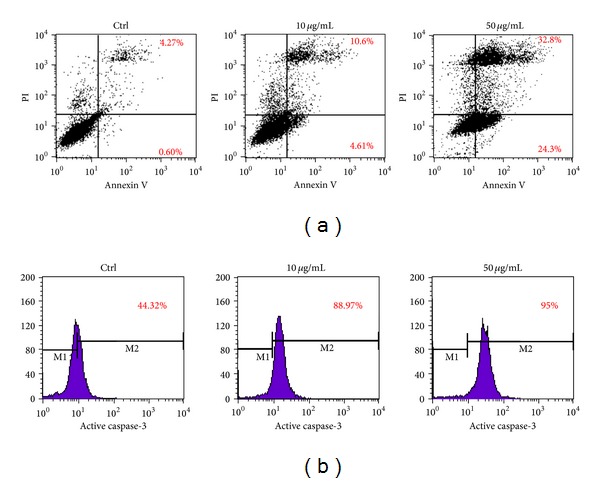
Apoptosis induction by jaboticaba seed water extract treatment of HSC-3 cells. (a) HSC-3 cells treated with jaboticaba seed water extract for 24 h at desired concentrations were stained with Annexin V-FITC and propidium iodide (PI). The Annexin V-FITC signal is shown on the* x-*axis and PI signal is shown on the* y*-axis. The apoptotic populations of cells were significantly increased in treatment groups in a dose-dependent manner. (b) Flow cytometric analysis was performed after a 48 h treatment to determine caspase-3 activity, which was found to be activated in a dose-dependent manner.

To further understand whether the decrease in cell numbers observed with the MTT assay was due to the slowdown of the cell cycle or the increase of apoptosis, we examined cell behavior after treatment using Annexin V/PI staining.  Figure 3(a) shows the Annexin V (+) population change after adding jaboticaba seed/water extract to HSC-3 cells. In the control group, the Annexin V (+) population was less than 5%. When HSC-3 cells were treated with the extract, the population increased to 15.2% and 57.1% in 10 and 50 *μ*g/mL treatments. In addition, the induction of caspase-3 activity was also examined by flow cytometry (Figure 3(b)). The increasing level of active caspase-3 with the elevated concentration of the extract showed that the apoptosis of HSC-3 cells was induced by the jaboticaba seed water extract.

### 3.4. Decrease of Survivin and Induction of Bid Cleavage Induced the Apoptosis Caused by Water Extracts of Jaboticaba Seeds

**Figure 4:**
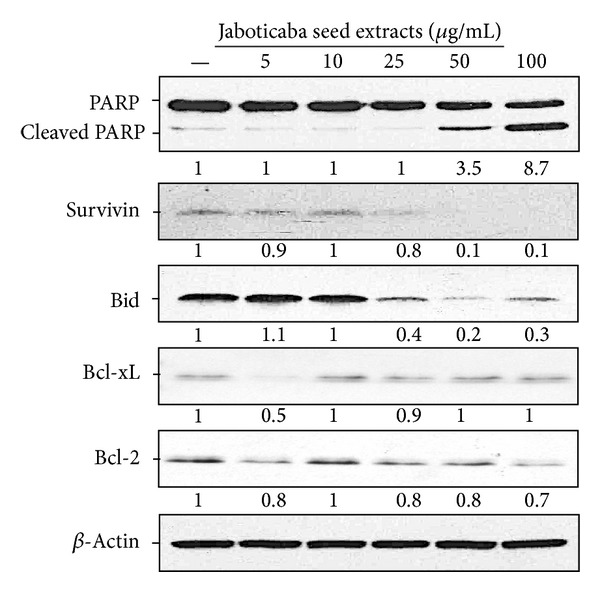
Effects of jaboticaba seed water extract on apoptosis regulatory proteins in HSC-3 cells. HSC-3 cells were treated with 5, 10, 25, 50, and 100 *μ*g/ml of jaboticaba seed extract or ddH_2_O as a control for 48 h, and then total proteins were isolated. Equal amounts of cell lysates were analyzed for PARP, survivin, Bid, Bcl-xL, and Bcl-2 expression by Western blotting with corresponding antibodies. *β*-actin served as the loading control. The fold change was calculated as the ratio of the target proteins in the presence of indicated concentration of Jaboticaba seed extract after normalization of the target proteins to *β*-actin in each lane.

Treatment of HSC-3 cells with different concentrations of water extracts of jaboticaba seeds for 24 h promoted dose-dependent cleavage of poly (ADP-ribose) polymerase (PARP) from the full length 116-kDa to an inactive 85-kDa form by activating caspases (Figure 4), which is another indicator of apoptosis. In order to further understand the apoptotic phenomenon, we evaluated the protein level of various key regulators in the apoptosis pathway through Western blot analysis. It is known that caspase-3 activity can be inhibited by a group of proteins that are collectively termed “inhibitors of apoptosis proteins,” of which survivin is one. We particularly determined the expression of survivin because it was shown to directly bind and inhibit caspase-3 [14, 15]. The dramatic abolishment of survivin thereby activated Bid cleavage, indicating that the water extract of jaboticaba seeds induced cell death by lowering the inhibition of apoptosis. Remarkably, the conventional intrinsic apoptosis pathway controlled by the Bcl-2 family did not show an unbalanced change.

## 4. Discussion

Jaboticaba has been reported to contain anthocyanins, flavonoids, phenolic acids, and tannins, which are well-known antioxidants with anti-inflammatory properties that are believed to play an important role in the prevention of certain diseases [2]. Traditionally, the jaboticaba fruit has been used as a treatment for hemoptysis, asthma, and diarrhea and gargled for chronic inflammation of the tonsils [4]. Recently, intake jaboticaba peels have been found to be able to attenuate oxidative stress in tissues and reduce circulating saturated lipids of rats with high-fat diet-induced obesity [23]. It was suggested that jaboticaba may have the potential to be developed as a functional food. Most studies have focused on the peel or the flesh extract of the fruit. However, the cancer chemopreventative activities of this fruit have not been extensively reported in the literature. In this study, extracts from different parts of jaboticaba were investigated for their antioxidant activity and water extract of jaboticaba seeds was further evaluated for antitumor activity against human oral cancer cell lines. Here we provide the first report revealing that the best antioxidant and cancer chemopreventative activity exists in the water-soluble seed extract.

In the present study, we examined different portions of jaboticaba including stem, peel, and seeds. The most notable cytotoxic activity against oral cancer cells appeared to be in seed extracts, particularly, water extracts. In terms of the extraction method, previous studies used methanol or ethanol as solvents to conduct extraction and, undoubtedly, the water extract composition will be changed as a result of the alcohol used. In addition, previous studies focused on analyzing the constituents of freeze-dried fruit, fresh fruit, peel, and pulp, but not seeds. Freeze-dried jaboticaba peels have been found to be rich in fiber and anthocyanins (delphinidin and cyanidin 3-glucoside) and showed high antioxidant activity. The jaboticaba peels extract showed antiproliferative effects against leukemic cells and prostate cancer cells [24]. Many phenolic constituents, like anthocyanins, were found to exist exclusively in the dark-colored fruit skin and not in the pulp or seeds [25, 26]. We are the first to demonstrate the appreciable anticancer activity of water extract of jaboticaba seeds. Purification and identification of the active compounds in the water extract of jaboticaba seeds are required for a better understanding of the protective mechanisms involved and to deduce possible applications in medicine.

Evasion of cell death is a characteristic feature of human cancers and represents a key source of resistance to current treatment approaches [27, 28]. Therefore, reactivation of cell death programs in cancer cells is a promising strategy to overcome resistance to treatment, which is one of the major unsolved problems in clinical oncology [29]. IAP proteins comprise a family of antiapoptotic proteins that promote prosurvival signaling pathways and prevent the activation of the effector phase of apoptosis by interfering with the activation of caspases [29]. Survivin, an IAP member [13], is an antiapoptotic protein that is basally expressed in normal tissue and overexpressed in nearly all human cancers. The expression of survivin in tumor cell lines increases with the proliferation rate and resistance to therapy [30]. Therefore, survivin is an emerging target for the development of novel anticancer therapies. The water extract of jaboticaba seeds induced oral cancer cell apoptosis by decreasing the expression of survivin. These results point to the potential of jaboticaba seed extract as a chemopreventive agent.

## 5. Conclusions

Dark-colored fruits like jaboticaba are a potentially rich source of many dietary phenolic antioxidants and are believed to play an important role in the prevention of many oxidative and inflammatory diseases. Most previous studies have focused on the alcohol extracts of flesh or peel. Here we showed that the water extract of jaboticaba seeds possesses appreciable antioxidant activity as well. Moreover, this is the first study to show the strong chemopreventative effects of the jaboticaba seed water extract. Our results show that the decrease of survivin and activated Bid cleavage is responsible for the ability of the jaboticaba seed water extract to induce apoptosis. Only a few studies have associated the consumption of jaboticaba with cancer protection. Based on these results, jaboticaba is promising not only as a source of antioxidants but also as a chemopreventative agent.
